# Development of a DNA marker for cultivar identification of “Harushizuka”, a new satsuma mandarin cultivar created by heavy-ion irradiation

**DOI:** 10.1270/jsbbs.25046

**Published:** 2026-02-13

**Authors:** Kotaro Ishii, Takahiro Okubo, Yuki Shirakawa, Kanako Tomura, Mitsuhiro Kato, Tsuyoshi Teraoka, Ikuo Sawano, Teruko Nakajima, Hiroshi Kagami, Akiko Kamio, Tomoko Abe

**Affiliations:** 1 RIKEN Nishina Center for Accelerator-Based Science, Wako, Saitama 351-0198, Japan; 2 Department of Radiation Measurement and Dose Assessment, Institute for Radiological Science, National Institutes for Quantum Science and Technology, Inage-ku, Chiba 263-8555, Japan; 3 Shizuoka Prefectural Research Institute of Agriculture and Forestry, Fruit Tree Research Center, Shizuoka, Shizuoka 424-0101, Japan; 4 Agricultural Strategy Division, Agriculture Bureau, Shizuoka Prefectural Government, Shizuoka, Shizuoka 420-8601, Japan; 5 Shizuoka Prefectural Research Center for Tea Science, Kikugawa, Shizuoka 439-0002, Japan; 6 Chuen Agricultural and Forestry Office, Shizuoka Prefectural Government, Iwata, Shizuoka 438-8558, Japan; 7 Shida-Haibara Agricultural and Forestry Office, Shizuoka Prefectural Government, Fujieda, Shizuoka 426-0075, Japan; 8 Fuji Agricultural and Forestry Office, Shizuoka Prefectural Government, Fuji, Shizuoka 416-0906, Japan

**Keywords:** *Citrus unshiu*, heavy-ion irradiation, whole-genome sequencing

## Abstract

‘Harushizuka’ is a novel late-maturing satsuma mandarin (*Citrus unshiu* Marcow.) cultivar developed through heavy-ion irradiation-induced mutagenesis. The S1152 line, selected from a nucellar seedling of the ‘Aoshima unshu’ cultivar for superior fruit quality, was irradiated with both carbon and neon ions. Mutations in fruit shape, color, and peel characteristics were observed in the neon-ion-irradiated group. From this group, we isolated the ‘Harushizuka’ cultivar, which exhibits delayed fruit coloring. The harvest season for ‘Harushizuka’ is approximately one month later than that of regular *C. unshiu*, allowing for a more spread-out harvesting period. To develop DNA markers for cultivar protection and identification, we performed whole-genome mutation analysis of ‘Harushizuka’ and its original cultivar ‘Aoshima unshu’. Two mutations were identified: a 30-bp deletion and a 20-bp insertion. The PCR assay targeting the deletion successfully differentiated ‘Harushizuka’ from 21 other *C. unshiu* cultivars. Our findings demonstrate that whole-genome mutation analysis is a powerful tool for developing DNA markers, even in citrus cultivars with low genetic diversity caused by bud mutations or nucellar embryogenesis. The established marker enables rapid and accurate identification of ‘Harushizuka’, contributing to the protection of breeders’ rights and the prevention of unauthorized propagation.

## Introduction

Satsuma mandarin (*Citrus unshiu* Marcow.) is the most widely produced fruit in Japan, with a cultivated area of 35,400 ha and a shipment of 617,100 tons in 2023 (MAFF 2024). In the same year, Shizuoka Prefecture had a cultivated area of 4,800 ha and shipped 90,600 tons, ranking third nationwide and making it one of the prefecture’s major agricultural products. Shizuoka Prefecture is a major production area for *C. unshiu*, primarily cultivating the ‘Aoshima unshu’ cultivar, which accounts for approximately 70% of the total *C. unshiu* cultivation area in the region. As a result, the concentration of harvest and shipping operations during a limited period, due to the predominance of a single cultivar, represents a major challenge for citrus production in the prefecture. More than 30 years ago, Shizuoka Prefecture selected two individuals (S1152 and S1164 lines) believed to be early-ripening strains from approximately 600 nucellar seedlings of ‘Aoshima unshu’. Although these strains exhibited an earlier harvest season compared to ‘Aoshima unshu’, these strains demonstrated poor fruit-bearing capacity, a tendency toward biennial bearing, and vigorous vegetative growth, which collectively hindered stable fruit production.

Over the past quarter-century, more than 90 new varieties and beneficial mutants have been developed in Japan using ion-beam irradiation (Abe 2021, Abe *et al.* 2012b, 2015). These mutants include rice with reduced cadmium accumulation (Ishikawa *et al.* 2012), aromatic rice with increased numbers of fertile grains (Okasa *et al.* 2020), tearless and non-pungent onion (Kato *et al.* 2016), lettuce with reduced polyphenol oxidase activity (Sawada *et al.* 2016), and peanuts with decreased levels of major allergens (Cabanos *et al.* 2012). Mutagenesis using heavy-ion beams has the advantage that a sufficient mutation rate can be achieved even at low doses that do not reduce survival rates, thereby enabling effective mutant selection (Ichida *et al.* 2019, Kanaya *et al.* 2008, Kazama *et al.* 2008, Oono *et al.* 2020).

In recent years, however, climate change has led to serious quality issues, such as an increased incidence of rind puffing and reduced citric acid content after long-term storage, both of which contribute to diminished fruit flavor. Rind puffing is a physiological disorder characterized by the significant separation of the peel from the flesh, resulting in reduced palatability and storability (Kawase *et al.* 1984). To address these challenges, we initiated a breeding program utilizing heavy-ion induced mutation techniques, resulting in the development of a novel, late-maturing cultivar of *C. unshiu*, ‘Harushizuka’. It was officially registered in Japan on March 6, 2024 (Registration No. 30107, https://www.hinshu2.maff.go.jp/vips/cmm/apCMM112.aspx?TOUROKU_NO=30107&LANGUAGE=Japanese).

Traditionally, cases of suspected infringement of breeders’ rights have been addressed by comparing the characteristics of trees and fruits. However, with recent advances in DNA analysis technology, simple and highly accurate methods for cultivar identification based on genetic information have been developed in plant science. This study aimed to establish a DNA-based identification method for ‘Harushizuka’, a cultivar expected to be widely adopted in Shizuoka Prefecture and to contribute to increased producer income. In citrus, several DNA marker systems have been developed and used, including SSR, CAPS (Fujii *et al.* 2019, Ninomiya *et al.* 2015, Nishimura *et al.* 2024, Nonaka *et al.* 2017), InDel markers (Noda *et al.* 2021, Okamoto *et al.* 2023), and TaqMan-MGB SNP genotyping assays (Endo *et al.* 2020). These markers are based on a combination of genotypes at multiple loci and offer the advantage of being immediately applicable to new varieties without the need for new markers. Additionally, they improve efficiency by requiring only a minimal set of markers. However, although these markers can be applied to hybrid varieties with high frequency, their application to varieties bred through genetic mutation is challenging (Noda *et al.* 2020). In cultivars developed through bud mutation, nucellar embryogenesis, or heavy-ion beam-induced mutations, genetic diversity is extremely low (Ichida *et al.* 2019, Kazama *et al.* 2017, Oono *et al.* 2020), making it difficult to detect polymorphisms and design DNA markers. To overcome this limitation, we employed next-generation sequencing (NGS) to analyze the whole genome of ‘Harushizuka’ and identify its unique mutation sites. NGS is a high-throughput sequencing technology that has significantly advanced research in the life sciences. In forward genetics, NGS plays a crucial role in gene function identification, as demonstrated in model plants such as *Arabidopsis thaliana* (Ashelford *et al.* 2011, Austin *et al.* 2011, Du *et al.* 2020, Katano *et al.* 2016, Nhat *et al.* 2021, Schneeberger *et al.* 2009, Uchida *et al.* 2011, Yamatani *et al.* 2018) and rice (*Oryza sativa*) (Abe *et al.* 2012a, Fekih *et al.* 2013, Koide *et al.* 2018, Morita *et al.* 2021).

In this study, we developed a new citrus cultivar, ‘Harushizuka’, through mutagenesis using heavy-ion irradiation and identified mutation sites unique to ‘Harushizuka’ through whole-genome analysis using NGS. Based on these results, we developed DNA markers that enable the accurate identification of ‘Harushizuka’.

## Materials and Methods

### Plant materials and heavy-ion mutagenesis

The S1152 line selected from the nucellar seedlings of ‘Aoshima unshu’ had an earlier ripening time than ‘Aoshima unshu’, but its fruit quality was as good as that of ‘Aoshima unshu’. The S1152 was used as the material for irradiation. The branches were cut to approximately 6 cm in length, arranged so that they did not overlap, and secured in place with adhesive tape so that the buds were aligned on the front. The axillary buds of branches were irradiated in the dose range of 10 to 50 Gy of neon (Ne) ions (135 MeV/u, 61 keV/μm) and 20 to 50 Gy of carbon (C) ions (135 MeV/u, 23 keV/μm) in the RI-Beam Factory from 2001 to 2002. Irradiated branches were grafted on 2-year-old *Poncirus trifoliata*. The survival rate was recorded 6 months after grafting the grown plants in the greenhouse. The survival plants (M_1_V_1_) were planted in the field in March 2004. Morphological mutants were selected in 2009, and from these, a secondary selection for agriculturally valuable traits was carried out in 2011. After that, investigations continued using M_1_V_2_ and M_1_V_3_ plants until 2019.

For cultivar identification using PCR, 22 citrus varieties and lines were used as test materials (Table 1). For each variety and line, hardened new leaves were collected from trees cultivated at the Fruit Tree Research Center of the Shizuoka Prefectural Research Institute of Agriculture and Forestry (Shizuoka City, Shizuoka Prefecture, Japan) for DNA extraction. Additionally, fruit peels were collected from seven of these varieties. All collected samples were stored either under refrigeration or frozen in liquid nitrogen until analysis.

### Whole-genome mutation analysis

Fresh leaves of ‘Aoshima unshu’ and ‘Harushizuka’ were flash-frozen in liquid nitrogen and ground into a fine powder. Genomic DNA was extracted using the DNeasy Plant Mini Kit (QIAGEN, Germany). The extracted DNA was sequenced using the HiSeq X Ten platform (Illumina, CA, USA). The resulting read sequences were analyzed using AMAP, as previously described (Ishii *et al.* 2016), with minor modifications: i) The pseudomolecule sequence of *C. unshiu* ‘Miyagawa wase’ (Shimizu *et al.* 2017), obtained from http://citrusgenome.jp (note that the website is currently inaccessible; the draft genome sequence is available at https://www.citrusgenomedb.org/Analysis/6389062, and the pseudomolecule sequence is accessible via the Wayback Machine at http://web.archive.org/web/20211127190709/http://citrusgenome.jp/), was used as the reference genome. From this genome, 390 sequences longer than 100 kbp were extracted and used for mutation analysis. ii) Among the software tools internally utilized by AMAP, only GATK (McKenna *et al.* 2010), Pindel (Ye *et al.* 2009), and BreakDancer (Chen *et al.* 2009) were used in this analysis. iii) Candidate mutations observed in both ‘Aoshima unshu’ and ‘Harushizuka’ were excluded as possible background mutations present in ‘Aoshima unshu’. iv) Candidate mutations were filtered by excluding those overlapping with pre-annotated repetitive regions (Shimizu *et al.* 2017), rather than identifying repetitive regions using RepeatMasker, as these were considered potential false positives. v) Candidate mutations with a read depth of less than 5 or greater than 1000 were excluded as potential false positives. vi) Candidate mutations supported by less than 20% of the reads mapped to the variant site were also excluded as potential false positives. The remaining candidate mutations were visually confirmed using the Integrative Genomics Viewer (IGV; Robinson *et al.* 2011) to ensure that they were not present in ‘Aoshima unshu’ and did not overlap with repetitive regions. For candidate mutations called by GATK, 300 sites were manually inspected after sorting them in descending order by quality score. We referred to the gene function prediction performed by Shimizu *et al.* (2017). To predict transcription factor binding sites around the mutation, we used the Binding Site Prediction tool of PlantRegMap (Tian *et al.* 2020), targeting a region 50 bp upstream and downstream of the mutation. The species was specified as *Citrus sinensis*.

### Cultivar identification using PCR

Hardened new leaves and fruit peels from each cultivar or line were rapidly frozen in liquid nitrogen and ground in microtubes. Genomic DNA was extracted from the resulting powdered samples using the DNeasy Plant Mini Kit (QIAGEN). The extracted DNA was stored at –20°C. Based on whole-genome mutation analysis, primers were designed to amplify mutation sites using Primer-BLAST (Ye *et al.* 2012), to generate PCR products of approximately 200 bp in length (Table 2). Genomic DNA was adjusted to a final concentration of 20 ng/μl and used as the template for PCR. The total volume of the PCR reaction mixture was 25 μl, consisting of the following components: 10 μl of sterile water, 12.5 μl of 2× Tks Gflex^TM^ Buffer (TaKaRa Bio Inc., Japan), 0.5 μl of Tks Gflex^TM^ DNA Polymerase (TaKaRa Bio Inc.), 1 μl of genomic DNA, and forward and reverse primers added to a final concentration of 0.5 μM each. PCR was performed under the following conditions: initial denaturation at 94°C for 1 min, followed by 36 cycles of 98°C for 10 s, 60°C for 15 s, and 68°C for 30 s, with a final extension at 68°C for 5 min. Amplified products were separated by electrophoresis using 4% MetaPhor Agarose (Lonza Japan Ltd., Japan) containing GelRed^TM^ (Biotium Inc., CA, USA) under conditions of 10 V for 22.5 h. Bands were visualized using the E-BOX gel documentation system (Vilber Bio Imaging, France).

### Data availability

The sequence data have been deposited in the DNA Data Bank of Japan Sequence Read Archive (DRA) under accession numbers DRR795572-DRR795573.

## Results

### Heavy-ion mutagenesis in *C. unshiu*

Survival rates decreased with increasing doses (Table 3). Many of the surviving branches exhibited abnormal leaf morphology in the first leaves after treatment, but they subsequently grew normally and produced normal leaves. When C and Ne ions were irradiated at 20 Gy or less, there were no differences in branch length and axillary bud number between wild-type and irradiated trees after 1 year (Kagami *et al.* 2003). The morphological mutations selected only from the Ne-ion irradiation treatment group in 2009 were leaf shape, fruit shape, smooth peel, chimaera peel, and delayed fruit coloring. In particular, morphological mutations in fruit appeared at the LD_50_ dose of 20 Gy (Table 3). Ultimately, the S1200 line, which exhibits delayed fruit coloring, was selected in 2011 from the 20 Gy Ne-ion irradiation treatment group. The shape of the harvested fruit of S1200 is almost indistinguishable from that of ‘Aoshima unshu’. However, the time when the fruit color changes from green to orange is one month later than that of ‘Aoshima unshu’. These characteristics were confirmed to be stable in experiments using M_1_V_2_ and M_1_V_3_ plants. In June 2021, S1200 was applied for cultivar registration as ‘Harushizuka’.

### Development of variety identification markers through whole-genome mutation analysis

**Fig. 1.**
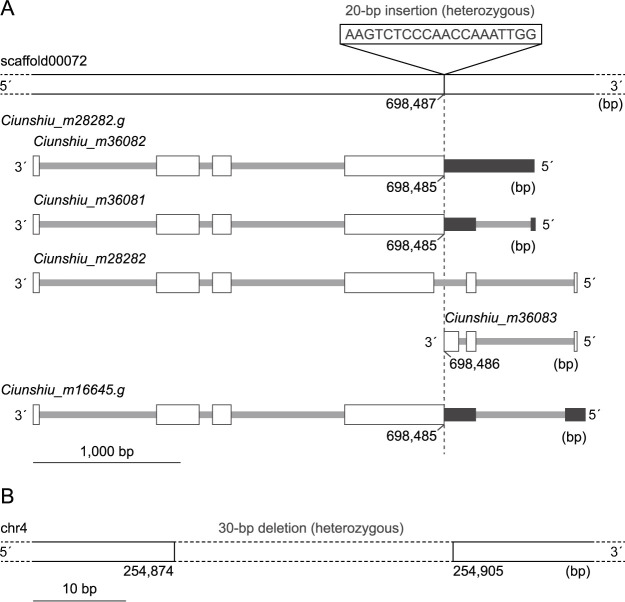
Two mutations identified in ‘Harushizuka’ through whole-genome mutation analysis. A. Insertion mutation on scaffold00072. B. Deletion mutation on chr4. White lines represent genomic sequences. White boxes represent coding sequences (CDSs), while black and grey lines indicate 5ʹ untranslated regions (5ʹ UTRs) and introns, respectively.

Although S1152 is the direct irradiated parent of ‘Harushizuka’, it has seen limited cultivation to date. In contrast, ‘Aoshima unshu’ is one of the most widely cultivated citrus varieties across Japan. Fruits harvested at similar maturity stages from ‘Aoshima unshu’ can be visually indistinguishable from those of ‘Harushizuka’, making it a likely candidate for misidentification. Therefore, from a practical standpoint, ‘Aoshima unshu’ was selected as a comparative variety to evaluate the effectiveness of molecular markers for the identification of ‘Harushizuka’. To detect mutations that occurred in ‘Harushizuka’, whole-genome sequencing was performed for both ‘Aoshima unshu’ and ‘Harushizuka’, yielding sequencing data equivalent to 211× and 198× genome coverage, respectively (Supplemental Table 1). The draft genome sequence of ‘Miyagawa wase’ (Shimizu *et al.* 2017) was used as the reference genome, and the analysis focused on large-scale structural variations to identify cultivar-specific markers, enabling cultivar identification using PCR. Mapping the read sequences from the two cultivars to the reference genome sequence, which was limited to sequences longer than 100 kbp, yielded average depths of 106 for ‘Aoshima unshu’ and 89.6 for ‘Harushizuka’ (Supplemental Table 1). Variant calling using GATK identified 18,155 candidate mutations excluding those located within repetitive sequences. These included small-scale mutations such as single-base substitutions. All of the candidate mutations were identified as being present in ‘Harushizuka’ and absent in ‘Aoshima unshu’. After sorting the candidate mutations by descending quality score, the top 300 were manually inspected. Although these mutations were initially classified as present in ‘Harushizuka’ but absent in ‘Aoshima unshu’, 246 were subsequently confirmed to be present in both cultivars. The remaining 54 sites were determined to be false positives (data not shown). As a result, no mutations specific to ‘Harushizuka’ were identified, possibly reflecting the low genetic diversity between ‘Aoshima unshu’ and ‘Harushizuka’. Next, we focused on indels, which are useful for mutation detection using PCR. 457 and 905 candidate mutations were identified by Pindel and BreakDancer, respectively, as being present in ‘Harushizuka’ but absent in ‘Aoshima unshu’. Manual inspection of these candidates led to the identification of two mutations: a 30-bp deletion located on chr4 of the reference genome and a 20-bp insertion located on scaffold00072 (Fig. 1). Since the proportion of reads supporting the variant at each site among the mapped reads is close to 50%, each variant was determined to be heterozygous (Table 4, Supplemental Figs. 1, 2). The insertion on scaffold00072 is identical to the subsequent 20 bp sequence and is therefore considered a tandem duplication (Supplemental Fig. 1B). A T-to-C substitution located 7 bp downstream of the insertion was also detected in ‘Aoshima unshu’ (data not shown), suggesting that this variant likely arose as a spontaneous mutation. This substitution is linked to the insertion. Although some reads carrying this substitution seem unlinked to the insertion, those of sufficient length have duplicated sequences that are soft-clipped at their ends, suggesting that the 20-bp sequence harboring the allele with this substitution was likely duplicated by heavy-ion beam irradiation. The insertion on scaffold00072 was found to affect two hypothetical genes, *Ciunshiu_m16645.g* and *Ciunshiu_m28282.g* (Fig. 1). Four transcript variants of *Ciunshiu_m28282.g* were predicted. In the variant *Ciunshiu_m36083*, the coding sequence (CDS) was disrupted by an insertion, resulting in a frameshift and the destruction of the stop codon, which may potentially affect the function of the encoded protein. The 5ʹ untranslated regions (5ʹ UTRs) were disrupted in the variants *Ciunshiu_m36081* and *Ciunshiu_m36082*. In the variant *Ciunshiu_m28282*, the insertion occurred in the second intron. In *Ciunshiu_m16645.g*, the 5ʹ untranslated region (5ʹ UTR) was disrupted. Genes with insertions affecting the CDS or the 5ʹ UTR exhibited sequence similarity to a gene encoding an E3 ubiquitin-protein ligase. Although the deletion on chr4 did not affect any gene (Fig. 1B), binding site prediction using PlantRegMap revealed that a C2H2 family transcription factor binding site (motif: orange1.1g011334m.g, p-value: 7.34e-05, q-value: 0.0158) was located within the deleted region (254,870–254,889 bp).

### Cultivar identification of ‘Harushizuka’ using PCR

**Fig. 2.**
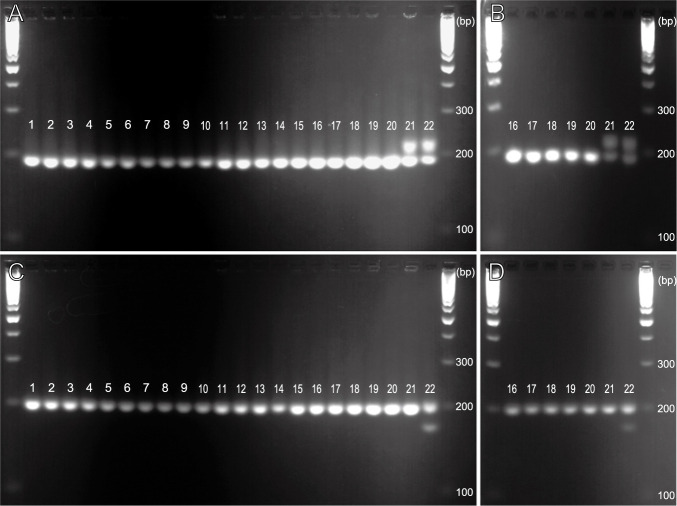
Cultivar identification of ‘Harushizuka’ using PCR targeting mutation sites. The lane numbers correspond to the cultivar or line numbers listed in Table 1. A–B. PCR results targeting the 20-bp insertion on scaffold00072. A. Genomic DNA extracted from hardened new leaves was used as the template. B. Genomic DNA extracted from fruit peels was used as the template. C–D. PCR results targeting the 30-bp deletion on chr4. C. Genomic DNA extracted from hardened new leaves was used as the template. D. Genomic DNA extracted from fruit peels was used as the template.

PCR primers were designed to target two mutations identified in this study (Table 2). PCR was performed using genomic DNA extracted from hardened new leaves of all 22 citrus cultivars or lines, and clear bands were observed in every case (Fig. 2A, 2C). To evaluate whether cultivar identification is feasible using fruit samples distributed in the market, fruit peels from seven of these cultivars were also used as PCR templates. These samples similarly yielded clear bands (Fig. 2B, 2D). The PCR targeting the insertion mutation on scaffold00072 could not distinguish between ‘Harushizuka’ and its irradiation source S1152, as both exhibited the same band pattern (Fig. 2A, 2B). This mutation is therefore not specific to ‘Harushizuka’ and is highly likely to have been present in S1152. In the PCR targeting the deletion mutation on chr4, only ‘Harushizuka’ produced a specific amplification product of 170 bp (Fig. 2C, 2D), which was not observed in the other 21 *C. unshiu* cultivars or lines, demonstrating its effectiveness for identifying ‘Harushizuka’. The 170-bp band specific to ‘Harushizuka’ appeared fainter than the upper band (Fig. 2C, 2D). One possible explanation is that the mutation exists as a chimera within the plant. However, this is unlikely, given that only 46% of the sequencing reads support the deletion (Table 4). An alternative possibility is that, in plants, the DNA methylation status of genomic regions can influence PCR amplification efficiency (Kiselav *et al.* 2015). These results indicate that the deletion mutation on chr4 serves as an effective DNA marker for distinguishing ‘Harushizuka’ from other *C. unshiu* cultivars.

## Discussion

In *C. unshiu*, the LD_50_ dose was found to be suitable for mutant selection. Even after irradiation at the LD_50_, one year later, no differences were observed in branch length or axillary bud number between wild-type and irradiated trees (Kagami *et al.* 2003). Heavy-ion beam irradiation allows for the selection of useful mutants at low doses without compromising survival rates. Examples of mutants that emerged at LD_50_ irradiation doses include dianthus mutants that produce white flowers (Sugiyama *et al.* 2008) and a microalgae mutant with an altered oil composition (Ikeda *et al.* 2014, Kaya *et al.* 2020). LD_50_ irradiation may be necessary for mutants that are difficult to produce. Mutations in the *C. unshiu* fruit were observed only after irradiation with 20 Gy of Ne ions with 61 keV/μm. Therefore, Ne ions with 61 keV/μm are more suitable than C ions with 23 keV/μm for *C. unshiu* mutagenesis by branches. Heavy-ion beams can change the LET (linear energy transfer, the amount of energy transferred per unit length to the material along the ion’s trajectory) by varying the ion species and ion velocity. In *A. thaliana*, the dose-response relationship for flowering rate was strongly dependent on the LET of the ions, rather than the ion species (Kazama *et al.* 2008). Furthermore, irradiation with C ions or Ar ions at 290 keV/μm resulted in high lethality (Kazama *et al.* 2008). Additionally, it exhibited similar effects on the frequency of morphological mutations and the characteristics of DNA alterations (Hirano *et al.* 2012). These findings suggest that the biological effects depend more on LET than on the ion species. As a result, C-ion irradiation induces only single-nucleotide variants (SNV) and deletions (DEL) with 23–30 keV/μm in rice mutants. In contrast, 50–60 keV/μm induces complex mutations accompanied by chromosomal structural changes such as insertions (INS), replacements (RPL), and inversions (INV) in addition to SNV and DEL. Ne-ion irradiation with 63–70 keV/μm also induces complex mutations similar to those induced by C ions with 50–60 keV/μm (Ichida *et al.* 2019). The fruit mutations were observed only with Ne-ion irradiation, which may suggest that complex mutations due to cluster damage occurring at 61 keV/μm are effective in inducing fruit mutations. In this study, mutation analysis revealed only two indel mutations, and no SNV mutations were detected. However, the absence of SNV mutations in this study does not necessarily mean that SNV mutations did not occur at all. To ensure the reliability of this method as a cultivar identification technique, candidate mutations located in repetitive sequence regions were excluded from the analysis. Furthermore, since the purpose of this paper is not to comprehensively examine all candidate mutations detected by GATK and discuss the overall mutation landscape, we focused on verifying only the top 300 most reliable candidate mutations.

‘Harushizuka’ is a mutant derived from S1152, which itself is a nucellar seedling of ‘Aoshima unshu’, generated through heavy-ion beam irradiation. In PCR targeting the deletion mutation on chr4, a single band was observed in 21 cultivars or lines, including S1152, while two bands were detected only in ‘Harushizuka’, indicating that this marker was likely created by heavy-ion beam irradiation. In contrast, PCR targeting the insertion mutation on scaffold00072 produced two bands in both ‘Harushizuka’ and S1152 among the 22 cultivars or lines tested. This mutation is not specific to ‘Harushizuka’ but is highly likely to have been present in S1152. Therefore, the same band was detected in both ‘Harushizuka’ and S1152, resulting in a distinct pattern from that of other *C. unshiu* cultivars. Although this is a common mutation found in both S1152 and ‘Harushizuka’, this insertion mutation on scaffold00072 is considered to affect genes that exhibited sequence similarity to a gene encoding an E3 ubiquitin-protein ligase. It has been reported that 1,415 genes of *A. thaliana* belong to the E3 ubiquitin ligase gene family (Mazzucotelli *et al.* 2006), accounting for approximately 5% of the total gene repertoire of this species. S1152 exhibits an earlier ripening phenotype compared to ‘Aoshima unshu’. In citrus, transcription factors belonging to the MADS-box family have been identified as regulators of fruit ripening (Terol *et al.* 2019). Although no direct interaction between MADS-box transcription factors and E3 ubiquitin ligases has been reported, E3 ubiquitin ligases are involved in regulating various biological processes in plants (Mazzucotelli *et al.* 2006); therefore, further investigation is required to determine how this mutation affects the phenotype.

‘Harushizuka’ exhibits a delayed fruit color change from green to orange, occurring approximately one month later than in ‘Aoshima unshu’, and the harvest time is also about one month later. A comparative transcriptome analysis between a late-ripening mutant and the wild type of *Citrus sinensis* identified 52 differentially expressed transcription factors. Among these, the C2H2 family was the third most abundant, as classified by Wu *et al.* (2016). In the present study, a deletion site on chr4 was found to contain a C2H2 family binding site, suggesting that transcription factor binding may be inhibited in ‘Harushizuka’. This mutation could potentially influence the delayed coloration phenotype; however, its exact role remains uncertain. In the mutation analysis, only the sequences longer than 100 kbp from the draft genome of ‘Miyagawa wase’ were used as the reference sequence. Notably, 7.3% of the short reads from ‘Harushizuka’ could not be mapped to this reference genome. The genes responsible for late-maturing traits in ‘Harushizuka’ may also be located in genomic regions that are not used as reference sequences.

In this study, we developed a DNA marker that enables the simple identification of the ‘Harushizuka’ cultivar using PCR. ‘Harushizuka’ is not only late-maturing, but also exhibits traits that make it suitable for long-term storage, such as a low incidence of rind puffing, high citric acid content, and is expected to be a variety that can be marketed in March or April (Sone *et al.* 2023). As the cultivation of ‘Harushizuka’ becomes more widespread, concerns arise about potential infringements of breeders’ rights. However, by utilizing the findings of this study, it will be possible to respond quickly to determine whether an infringement has occurred. Furthermore, this technology is expected to act as a deterrent against cultivar theft, thereby strengthening the protection of breeders’ rights.

## Author Contribution Statement

KI, TO, HK, and TA conceived and designed the study. TT, IS, TN, HK AK, and TA experimented with mutant screening. KI and YS conducted the whole-genome mutation and sequence analyses. TO, KT, MK, and YS performed the PCR experiments. KI, TO, and TA wrote the manuscript. All authors read and approved the final version of the manuscript.

## Supplementary Material

Supplemental Figures

Supplemental Table

## Figures and Tables

**Table 1.** Cultivars and lines used for cultivar identification

No.	Variety or line	Scientific name	Registration No.*^b^*	JP NO.*^c^*	Origin/Mutation type
1	‘Nichinan No. 1’	*Citrus unshiu* Marcow.	1981	—	Bud mutations of ‘Okitsu wase’
2	‘Ueno wase’		915	—	Bud mutations of ‘Miyagawa wase’
3	‘Yura wase’		4723	—	Bud mutations of ‘Miyagawa wase’
4	‘Toyofuku wase’		4423	—	Nucellar seedling derived from ‘Ooura wase’
5	‘Miyamoto wase’		82	—	Bud mutations of ‘Miyagawa wase’
6	‘Takabayashi wase’		637	—	Mutations of ‘Okitsu wase’
7	‘Miyagawa wase’		—	117351	Bud mutations of original satsuma
8	‘Okitsu wase’		Mikan Norin No. 1	170630	Nucellar seedling derived from ‘Miyagawa wase’
9	‘Taguchi wase’		4725	—	Bud mutations of ‘Okitsu wase’
10	‘Yamashita beni wase’		410	—	Bud mutations of ‘Miyagawa wase’
11	‘Otsu No. 4’		312	117319	Nucellar seedling derived from ‘Juman unshu’
12	‘Ishiji’		8449	—	Mutations of ‘Sugiyama unshu’
13	‘Sugiyama unshu’		—	117516	Mutations of Owari-line *Citrus unshiu*.
14	‘Katayama unshu’		—	168850	Mutations of Owari-line *Citrus unshiu*.
15	‘Imamura unshu’		206	117329	Mutations of Owari-line *Citrus unshiu*.
16*^a^*	‘Aoshima unshu’		—	117320	Bud mutations of Owari-line *Citrus unshiu*.
17*^a^*	A-44		—	—	Gamma ray-induced mutant derived from ‘Aoshima unshu’
18*^a^*	‘Jutaro unshu’		642	—	Bud mutations of ‘Aoshima unshu’
19*^a^*	‘Mineta’		23035	—	Mutations of ‘Aoshima unshu’
20*^a^*	‘Yoichiro’		20680	—	Bud mutations of ‘Aoshima unshu’
21*^a^*	S1152		—	—	Nucellar seedling derived from ‘Aoshima unshu’
22*^a^*	‘Harushizuka’		30107	—	Ion beam-induced mutant derived from S1152

*^a^* Both hardened new leaves and fruit peels were collected. *^b^* Description by the Plant Variety Protection Office, MAFF, Japan (https://www.hinshu2.maff.go.jp/en/en_top.html). *^c^* Description by the Genebank at NARO, Japan (https://www.gene.affrc.go.jp/databases-plant_search.php).

**Table 2.** PCR primers for the identification of the ‘Harushizuka’ cultivar

Target		Primer sequence (5ʹ to 3ʹ)	PCR amplicon size (bp)	
Scaffold	Mutation		‘Aoshima unshu’	‘Harushizuka’
scaffold00072	20-bp insertion	forward: GCGATGCACCTGCTCCATTA reverse: AGATGTTTGTTCACAGAGGGGA	193	193 + 213
chr4	30-bp deletion	forward: AGCACATGATATGAAGTACGCT reverse: ACCACATTGGAGTAGTGACTGT	200	200 + 170

**Table 3.** Morphological mutants (M_1_V_1_) induced by heavy-ion irradiation

Ion	Dose (Gy)	No. of irradiated branches	No. of survival plants (%)	No. of morphological mutants	Characteristics of mutants
C	20	60	20 (33)	0	
	30	20	8 (40)	0	
	40	20	3 (15)	0	
Ne	10	40	24 (60)	1	Leaf: obovate shape
	20	60	29 (48)	4	Fruit: delayed fruit coloring, smooth peel, chimeric peel, oblate shape
	50	20	3 (15)	0	

**Table 4.** Mutations uniquely identified in the ‘Harushizuka’ cultivar

Location			Mutation	Zygosity	Mutation read proportion (%)*^a^*	Mapped read count*^a^*
Scaffold	Start	End				
chr4	254875	254904	30-bp deletion	heterozygous	49	39
scaffold00072	698487	698488	20-bp insertion	heterozygous	46	96

*^a^* Value at start location.
