# Supplementary material for: Development of a DNA marker for cultivar identification of “Harushizuka”, a new satsuma mandarin cultivar created by heavy-ion irradiation
Source: Breed Sci. 2026 Feb 13;76(1):111–9. doi: 10.1270/jsbbs.25046 (PMC13437163; doi:10.1270/jsbbs.25046)
Supplement: Supplementary file 1 [file 76_111_s1.pdf]

doi: 10.1270/jsbbs.25046

A

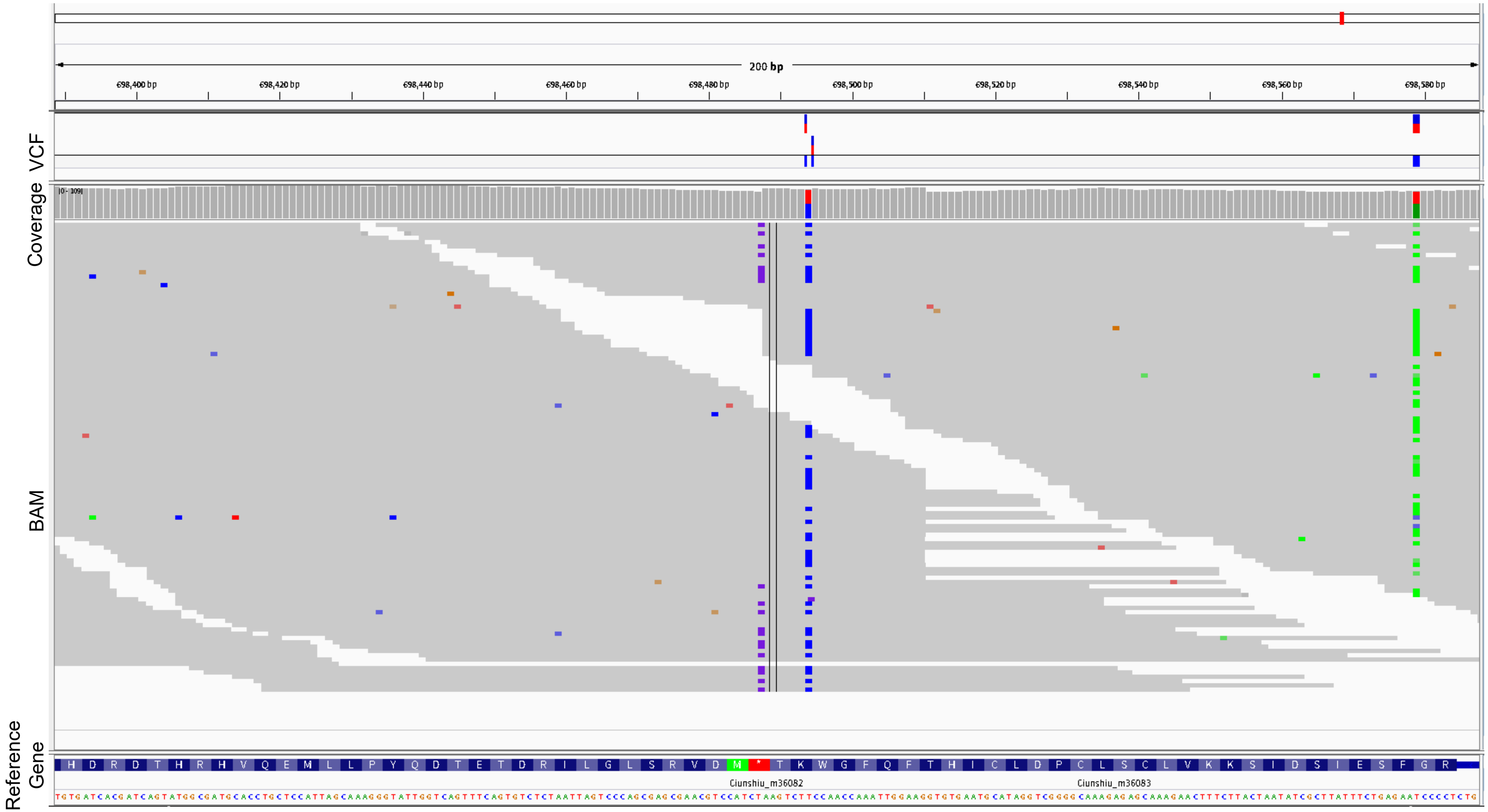

B

Pindel: CATCT [AAGTCTCCCAACCAAATTGG] [AAGTCTCCCAACCAAATTGG] AAGGT

GATK: CATCT [AAGTCTCCCAACCAAATTGG] [AAGTCTCCCAACCAAATTGG] AAGGT

Supplemental Figure 1. A. Read alignment around the insertion detected on scaffold00072 in the cultivar 'Harushizuka' visualized using IGV. In the mapped reads, regions identical to the reference sequence are shown in gray, while specific colors indicate base substitutions: green for A, red for T, brown for G, and blue for C. Insertions are represented by purple bars. In Pindel, the insertion was identified as a 20 bp insertion at position 698,487, but in the GATK-generated VCF file, it was split into two separate events. The first apparent insertion (CCCAACCAAA) at position 698,493 has variant attributes including Allele Count (1), Allele Frequency (0.5), Mapping Quality (60.00), and Depth (91), while the second apparent insertion (TGGAAGTCTC) at position 698,494 has variant attributes including Allele Count (1), Allele Frequency (0.5), Mapping Quality (60.00), and Depth (89). B. Differences in insertion detection results between Pindel and GATK. The nucleotide sequence from positions 698,483 to 698,512 on scaffold00072 is shown. Bases in white on a black background indicate inserted sequences. Bases enclosed in brackets represent one unit of duplication. The base in red indicates a T-to-C nucleotide substitution, which was also detected in 'Aoshima unshu', suggesting that this is likely a spontaneous mutation.

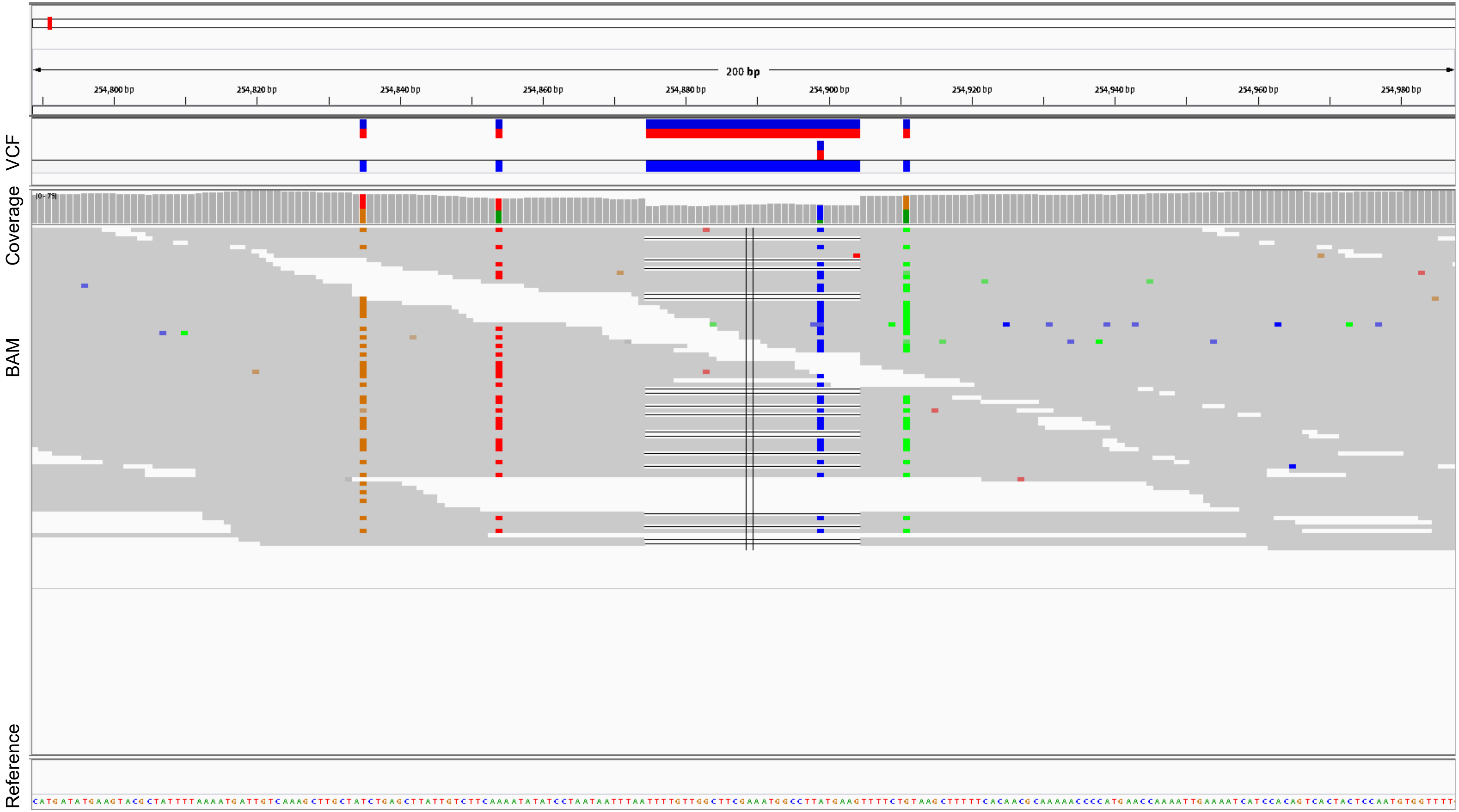

Supplemental Figure 2. Read alignment around the deletion detected on chr4 in the cultivar 'Harushizuka', visualized using IGV. In the mapped reads, regions identical to the reference sequence are shown in gray, while specific colors indicate base substitutions: green for A, red for T, brown for G, and blue for C. Black lines represent the deleted regions. In the VCF file generated by GATK, the deletion has variant attributes including Allele Count (1), Allele Frequency (0.5), Mapping Quality (60.32), and Depth (68).
