# Supplementary material for: Development of a DNA marker for cultivar identification of “Harushizuka”, a new satsuma mandarin cultivar created by heavy-ion irradiation
Source: Breed Sci. 2026 Feb 13;76(1):111–9. doi: 10.1270/jsbbs.25046 (PMC13437163; doi:10.1270/jsbbs.25046)
Supplement: Supplementary file 2 [file 76_111_s2.pdf]

Ishii et al./Breeding Science
doi: 10.1270/jsbbs.25046

Supplemental Table 1. Summary of genome sequencing.

Cultivar	Data volume (Gbp)	Genome coverage	Average depth
'Aoshima unshu'	75.9	211×	106
'Harushizuka'	71.1	198×	89.6
